# Cellular therapy for the peripheral arterial disease treatment: Protocol for a systematic review and meta-analysis

**DOI:** 10.1371/journal.pone.0314070

**Published:** 2025-01-09

**Authors:** Mónica Rodríguez-Valiente, Cristina Fuente-Mora, Javier Sánchez-Gálvez, Micaela Ortín-Pérez, Manuel Pardo-Ríos, José Manuel Sánchez-Sáez, Isac Davidson Santiago Fernandes Pimenta, Grasiela Piuvezam, Laura Ramos-Petersen

**Affiliations:** 1 Catholic University San Antonio de Murcia, Murcia, Spain; 2 Biomedical Research Institute of Murcia (IMIB), Murcia, Spain; 3 Federal University of Rio Grande do Norte and Systematic Review and Meta-Analysis Laboratory (LabSys-CNPq), Natal, Brazil; 4 Department of Nursing and Podiatry, University of Malaga, Malaga, Spain; Hiroshima University: Hiroshima Daigaku, JAPAN

## Abstract

Cellular therapy is a promising treatment option for Peripheral Arterial Disease (PAD). Different cell types can be used to regenerate and repair tissues affected by PAD. Many studies have proposed the use of stem cells, such as mesenchymal stem cells, or even mononuclear cells isolated from peripheral blood or bone marrow, to treat PAD. This paper reports a protocol of systematic review and meta-analysis that aims to identify the effects of stem cell treatment in patients with peripheral arterial disease. The search will be conducted in the following database: PubMed/MEDLINE, Clinicaltrial.gov, Scopus, Embase, Epistemonikos, Web of Science and Cochrane Library. Studies will be selected independently by two reviewers and will include all published randomized and non-randomized clinical trials. The data extraction will include studies population characteristics, type of treatment and main outcomes. We will assess the methodological quality of the studies using the Cochrane Risk of Bias 2.0 and Risk of Bias in Non-randomized Studies of Interventions. The certainty of the evidence will be rate using the Grading of Recommendations, Assessment, Development, and Evaluations. The findings will be presented in narrative summary tables and in a meta-analysis.

## Introduction

Chronic Limb Threatening Ischemia (CLTI), also known as Critical Limb Ischemia (CLI), is characterized by chronic pain at rest, ulcers, and gangrene due to arterial occlusive disease [1]. This condition often leads to amputation of the lower extremities and can be fatal. CLTI is particularly prevalent in diabetic patients, presenting a significant health burden due to the limited treatment options available to slow its progression [2, 3].

Patients with Peripheral Artery Disease (PAD) are at high risk of developing CLTI, which can result in lower limb amputation and high mortality rates [4]. Approximately 30% of patients undergoing leg amputation for atherosclerotic PAD die within a year, and the 5-year survival rate is less than 30% [5]. Despite the severe social and health impacts, treatment options for these patients remain limited [6].

Surgical revascularization, including angioplasty, stents, and bypass grafting, is often the treatment of choice. However, these invasive procedures come with high complication rates and are not always successful in resolving ischemia. Atherosclerotic relapses, hyperplasia of the tunica intima and media, and postoperative thrombosis necessitate arterial reintervention in 30–50% of operated patients [7]. Additionally, many patients are not suitable candidates for surgery due to the distal location of obstructions or the presence of comorbidities that increase surgical risk.

Recent research has focused on therapeutic angiogenesis to improve tissue perfusion in ischemic tissues. This approach includes the administration of recombinant growth factors [8–11] and gene therapy using vectors to promote the proliferation of collateral vessels [12]. Mesenchymal stem cells (MSCs) have shown broad therapeutic potential due to their regenerative capacity, ease of isolation, and low immunogenicity [13]. MSCs can differentiate into various cell types, including neurons, lung epithelial cells, pancreatic beta cells, corneal epithelial cells, and cardiomyocytes, making them suitable for treating numerous damaged tissues and degenerative disorders [14–19].

Recent advancements in cell-based therapies show significant potential in the treatment of chronic ulcers. One approach involves the use of adipose-derived stem cells (ADSCs), which have been applied to treat persistent skin ulcers. These cells either differentiate into specific cell types or produce various angiogenic growth factors, such as basic fibroblast growth factor, platelet-derived growth factor, and vascular endothelial growth factor, aiding in tissue repair [20].

The Therapeutic Angiogenesis using Cell Transplantation (TACT) study provided further evidence by showing that intramuscular injection of autologous bone marrow mononuclear cells (ABMNCs) into critically ischemic legs increased perfusion and improved clinical outcomes [21]. Research indicates that postnatal neovascularization involves not only the migration and proliferation of resident endothelial cells but also the differentiation of EPCs derived from bone marrow. Peripheral blood contains EPCs capable of maturing into fully functional endothelial cells, contributing to neovascularization [22].

Given the complications associated with PAD and the challenges in achieving successful treatment, it is crucial to systematically review the advances in cell-based therapies. This paper reports a protocol to a systematic review and meta-analysis aiming to assess the efficacy and limitations of various angiogenic cell therapies in treating patients with PAD, providing a comprehensive overview of current progress and future directions.

## Methods

### Study registration

The protocol of this study was previously registered on the International Prospective Register of Systematic Reviews (PROSPERO) database (CRD42022339988) and developed in accordance with the Preferred Reporting Items for Systematic Review and Meta-analysis Protocols (PRISMA-P) guidelines (S1 File) [23]. We will use the PRISMA [24] statement and the Cochrane Handbook for Systematic Reviews of Interventions [25] as guidance to report the systematic review.

### Review question

Our main research question is: What are the effects of stem cell treatment compared to standard treatments (gold standard) in patients with peripheral arterial disease? We also have two secondary questions: What kind of administration of stem cells is more used in clinical trials? Which tissues are obtained in these studies?

### Eligibility criteria

#### Participants

The studies included in this review will be clinical trials enrolling adults (aged 18 and over) of both sexes with peripheral artery disease caused by atherosclerosis who previously underwent a non-effective standard vascular treatment.

#### Interventions

We will consider interventions which include treatments with the following cells: In particular, mesenchymal stem cells (CD 34-, CD45-, CD90+, CD 73+, CD44+), hematopoietic stem cells CD 34+ and mononuclear cells extracted from adipose tissue and/or bone marrow or peripherally blood and are autologous or allogeneic. The administration route can be one of the following options: Intramuscular (IM), Intravenous (IV), Intra-arterial (IA) Intradermal (ID).

#### Comparison

Studies with or without control group that compare the intervention with an alternative or standard treatment will be considered.

#### Outcomes

The outcome measures will consider the non-progression of the disease/beneficial effects with the purpose of neovascularization (neoangiogenesis or formation of collateral vessels).

As additional outcomes, ankle-brachial index, finger-brachial index, angiography, angio-resonance imaging, Magnetic Resonance Imaging, percutaneous oxygen, visual analogue scale, Rutherford Becker and Fontaine Scale, Wound ischemia foot infection (WIfI) ulcer evaluation and planimetry will be considered.

#### Types of studies

Randomized and non-randomized clinical trials will be included.

### Information sources and search strategies

The search will be performed in the following databases: PubMed/MEDLINE, Clinicaltrial.gov, Scopus, Embase, Epistemonikos, Web of Science and Cochrane Library. Combinations between the terms from the Medical Subject Headings (MeSH) and keywords: stem cells, Cell- and Tissue-Based Therapy and peripheral arterial disease will be used to guarantee a broad research strategy, according to the characteristics of each database, accompanied by the Boolean operators “AND” and “OR”. The search strategy to the PubMed/MEDLINE database is available as support information to this paper (S2 File). The search terms will be combined with the specific filters in each database. No restrictions to language or year of publication will be set.

### Study selection

Following the retrieval of records from all databases, an initial examination will be conducted to identify any duplicates, which will then be appropriately removed using the Rayyan QCRI tool. Two independent reviewers will screen the database records, reviewing titles and abstracts. The studies that are selected will be fully read to ensure they meet the eligibility criteria.

Subsequently, two reviewers will examine the full text of all studies that meet the eligibility criteria. The consensus between the reviewers will be evaluated using the Kappa index. In the event of any disagreements between reviewers at any stage of the study selection process, a third reviewer will be consulted to resolve the issue. We will also conduct a manual search in the references of the included studies.

### Data extraction and management

Two reviewers will independently extract the data using a previously tested standard electronic spreadsheet. The extracted data will encompass the identification details of the studies (including the year, primary author, country, and type of clinical trial), characteristics of the population (such as age, sex, and condition), the type of treatment administered, and the outcomes. In cases where a result is measured at various time points, the mean values and standard deviation will be incorporated.

### Dealing with missing data

In the event of missing or unclear data, the research team will attempt to contact the corresponding author or co-author by e-mail. If this communication is unsuccessful, the data will be excluded from the analysis, and we will address this in the discussion section.

### Risk of bias and quality assessment

Two reviewers will independently assess the included studies using the Cochrane Risk of Bias version 2 (ROB2) for randomized designs and the Risk Of Bias in Non-randomized Studies of Interventions (ROBINS-I) for non-randomized designs. They will categorize the risk of bias as either low, high, or unclear [25]. Disagreement in the assessment will be solved by a third reviewer.

### Data synthesis

Data will be presented in summary tables and in narrative form to describe the characteristics of the included studies. Indicators of care processes and results will be analysed separately. If the included studies were sufficiently homogeneous, we will perform a quantitative synthesis of the results.

The results obtained in the included studies will be extracted to calculate the delta of variation (o) and standard deviations of variation for the intervention and control groups. Then, in the comparative analyses the results will be presented through the standardized mean differences (SMD) between the groups (intervention and control) for each included study.

To perform the meta-analysis, Review Manager 5.3 will be used (The Nordic Cochrane Centre: Copenhagen, Denmark) [26], a software recommended by the Cochrane Handbook for Systematic of Interventions [25]. The random effects model will be used to calculate the total effect size of the studies included in the meta-analysis.

The evaluation of heterogeneity between studies will be verified by chi-square test (X^2^) with a significance level (p< 0.05) and I^2^ statistical tests. I^2^ assesses the proportion of variability between studies and levels that can be classified into low levels (0% to 25%), medium levels (above 25% to 50%), or high levels of heterogeneity (above 50%). If substantial heterogeneity occurs, we will perform subgroup analysis and meta-regression to identify possible associated cofactors by sex, severity of arterial obstruction, type of cellular therapy, and risk of bias.

If possible, funnel plots will be used to assess the presence of potential reporting biases. A linear regression approach will be used to evaluate funnel plot asymmetry.

### Assessment of quality of evidence

Two independent reviewers will rate the certainty of the evidence provided by the selected studies using the Grading of Recommendations Assessment, Development and Evaluation (GRADE) [27]. The quality of evidence will be classified into four levels (high, moderate, low and very low) and the strength of the evidence into two levels (strong or weak)

### Dissemination and ethics

The findings from the systematic review will be published in a peer-reviewed journal. Additionally, they will be shared within academic and health services contexts, including conferences and seminars. No ethical committee approval is necessary since the review does not involve the collection of personal data from professionals or patients.

## Discussion

Treatment options for patients with severe PAD for whom conservative management has failed and who are not candidates for surgical interventions, such as endovascular or open procedures, are sparse. Such patients typically must resort to limb amputation as a final option. Beginning in 2000, several animal model studies reported successful outcomes using stem cell therapy to improve peripheral blood circulation [28–30].

The goal of this type of cell therapy is to promote neoangiogenesis, by increasing circulation, reducing symptoms, and facilitating wound healing in patients with PAD. This is achieved by administering different types of angiogenesis progenitors’ cells.

Throughout the procedure, the role of the team that performs the process is crucial, from the extraction and collection of cells at the indicated time to the administration and subsequent control and monitoring of the patient. During this process, it is essential the coordination of the team made up of laboratory specialists, doctors and nursing staff that work together for an optimal result.

Despite the promise shown by cell therapy in improving peripheral blood circulation for PAD patients, some challenges remain to this future review. There is a scarcity of large-scale clinical trials focused on cell therapy in patients with PAD, which leads to small sample sizes and reduces the statistical power of studies. We also may find a high heterogeneity between studies, as different types of angiogenesis progenitor cells are used, and variations in study design, patient characteristics, and outcome measures exist.

Still, the review will provide valuable information to reverse or mitigate the damage experienced by patients afflicted with this severe disease, helping professionals to select more effective treatment options. It will also benefit the scientific community, providing a summary of the current knowledge about this treatment and the limitations that require the development of further research.

## Supporting information

S1 File PRISMA-P checklist. (DOC)

S2 File PubMed/Medline search strategy. (DOCX)
