# Supplementary material for: Cellular therapy for the peripheral arterial disease treatment: Protocol for a systematic review and meta-analysis
Source: PLoS One. 2025 Jan 9;20(1):e0314070. doi: 10.1371/journal.pone.0314070 (PMC11717238; doi:10.1371/journal.pone.0314070)
Supplement: S2 File — (DOCX) [file pone.0314070.s002.docx]

**S2 File. PubMed/Medline search strategy.**

**#1** ("Stem Cells"[Mesh]) OR (Cells, Stem) OR (Cell, Stem) OR (Stem Cell) OR (Mother Cells) OR (Cell, Mother) OR (Cells, Mother) OR (Mother Cell) OR (Progenitor Cells) OR (Cell, Progenitor) OR (Cells, Progenitor) OR (Progenitor Cell) OR (Colony-Forming Unit) OR (Colony Forming Unit) OR (Colony-Forming Units) OR (Colony Forming Units)

**#2** ("Cell- and Tissue-Based Therapy"[Mesh]) OR (Cell and Tissue Based Therapy) OR (Cell Therapy) OR (Therapy, Cell) OR (Tissue Therapy) OR (Therapy, Tissue)

**#3** = **#1** OR **#2**

**#4** ("Peripheral Arterial Disease"[Mesh]) OR (Arterial Disease, Peripheral) OR (Arterial Diseases, Peripheral) OR (Disease, Peripheral Arterial) OR (Diseases, Peripheral Arterial) OR (Peripheral Arterial Diseases) OR (Peripheral Artery Disease) OR (Artery Disease, Peripheral) OR (Artery Diseases, Peripheral) OR (Disease, Peripheral Artery) OR (Diseases, Peripheral Artery) OR (Peripheral Artery Diseases)

**#5** = **#3** AND **#4**
